# The Level of Selected Bacterial Phyla on the Skin Surface of Small Ruminants According to the Breed and Species

**DOI:** 10.3390/ani11092734

**Published:** 2021-09-18

**Authors:** Paulina Cholewińska, Paulina Nazar, Andrzej Junkuszew, Jakub Smoliński, Katarzyna Czyż, Anna Wyrostek

**Affiliations:** 1 Institute of Animal Breeding, Wrocław University of Environmental and Life Sciences, 51-630 Wrocław, Poland; 107848@student.upwr.edu.pl (J.S.); katarzyna.czyz@upwr.edu.pl (K.C.); anna.wyrostek@upwr.edu.pl (A.W.); 2 Department of Animal Breeding and Agricultural Advisory, University of Life Sciences in Lublin, 20-950 Lublin, Poland; paulina.dudko@up.lublin.pl (P.N.); andrzej.junkuszew@up.lublin.pl (A.J.)

**Keywords:** sheep, goats, skin, microbiome, Uhruska sheep, Świniarka sheep, BCP line, Sanaan goat, Boer goat

## Abstract

**Simple Summary:**

The skin is one of the largest surface organs for animals. The microbiome of the skin plays an important role in protecting the host. The study showed that the environment in which the animals lived and their size could affect the bacterial composition of their skin. Additionally, individual differences between the bacterial composition of the skin were observed, which may indicate the existence of a factor called “individual influence”.

**Abstract:**

For decades, skin has been assigned the main role of an insulator of the inside of the body from the external environment, but it also plays a role in maintaining homeostasis. In this study, the level of selected bacterial phyla (Firmicutes, Bacteroidetes, Proteobacteria and Actinobacteria) was assessed in three sheep breeds (Świniarka sheep, Uhruska sheep and BCP line (synthetic sheep breed; *n* = 6) and in two breeds of goats (Boer, Saenian; *n* = 6) living in the same environment and fed on the same feed, where the aim was to identify differences in terms of race, species and individual differences. Significant differences were found in Firmicute, Actinobacteria and Proteobacteria phyla (*p* ≤ 0.05). Statistically significant and positive correlations were demonstrated between Actinobacteria and Proteobacteria, Proteobacteria and Bacteroidetes or Firmicutes and Bacteroidetes. The obtained results suggest that the species and racial differences in the level of the studied bacterial phyla may also result from the physicochemical differences of the skin surface, as they could exacerbate the variations in humidity, temperature, composition of antimicrobial peptides (AMP) and lipid content. In addition, individual differences were observed, which indicate a similar effect of an individual on the microbiological composition of its organism.

## 1. Introduction

The skin of vertebrates is the largest organ in terms of surface area. For decades, it was mainly assigned the role of an insulator separating the internal system of animals from the external environment. However, many studies have shown that the skin plays many key roles in maintaining the body’s homeostasis. It acts as a protective barrier thanks to both ectoderm cells and microorganisms on its surface. Microorganisms on the surface of the skin form a specific type of ecosystem, the main purpose of which is to protect against the colonization of pathogens and to maintain the skin in a proper condition. Both the quantity and the variety of microorganisms are important issues, and the disorder, i.e., the occurrence of dysbiosis, may be influenced by factors such as nutrition or environmental conditions (temperature, humidity and sunlight), and thus the conditions of maintenance [1,2,3]. In mammals, the most numerous groups of bacteria belong to the Firmicutes and Bacteroidetes types; although less numerous, the most common are Actinobacteria and Proteobacteria. However, their occurrence is largely on the skin, among other things, depending on the breed of animals, species or the place where they live [4].

It is estimated that around 3.9 billion ruminants are kept currently using sustainable agricultural practices, allowing, among others, the use of uncultivated land through grazing, the use of industrial by-products as a source of food and the production of energy from low-quality forage obtaining high quality products—milk, wool and meat. Therefore, for ruminants, both the digestive system and the skin microbiome (related to the production of wool or leather) are important. It should be taken into account that the skin, in case of improper maintenance conditions or diet, will often show any abnormalities or changes occurring in the body [5,6,7]. In recent years, apart from environmental factors, genetic factors (including even individual ones) that may affect the animal microbiome are also taken into account—which is also important for the breeder to react faster to abnormalities in the animal’s health. However, this type of research is performed mainly in terms of the variability of the digestive system microbiome. Attention should be also paid to the condition of the skin microbiome, which is just as important as the digestive or reproductive system microbiome.

The microbiome on the skin surface consists of bacteria, protozoa, fungi and viruses, the number of which is very variable, due to many factors that may affect its composition, both quantitative and qualitative. The skin microbiome, similar to the digestive system, can modulate the innate and adaptive immune response of the skin, which in turn helps the body educate the immune system [3]. The occurrence of skin dysbiosis can alter the production of metabolites and increase the risk of inflammation [6,7].

In this study, the level of selected bacterial phyla (Firmicutes, Bacteroidetes, Proteobacteria and Actinobacteria) was assessed in three breeds of sheep and in two breeds of goats living in the same environment and fed the same feed, and the aim was to determine the differences in terms of breed, species and individual.

## 2. Materials and Methods

### 2.1. Animals

Three breeds of sheep and two breeds of goats (*n* = 6 for each group) were included in the experiment:

1. Świniarka sheep: primitive breed, small, fine, mixed wool; ewes’ weight: 25–35 kg, seasonal [8].
2. Uhruska sheep: medium-sized, closed woolly cover; ewes’ weight: 55–80 kg [8].
3. BCP line (Polish lowland sheep 37.5%, fertile breed (Romanov, Friesian, Finnish) 12.5%, Berrichon 25.00% and Charolaise 25.00%): large, well-defined muscles; ewes’ weight: 50–70 kg [8].
4. Saanen goat: large framed, short, white and shiny hair, dairy breed; body weight of goats: 50–90 kg [8].
5. Boer goat: short hair, white and shiny, compact and stocky body; body weight of goats: 50–90 kg [8].

**Figure 1:**
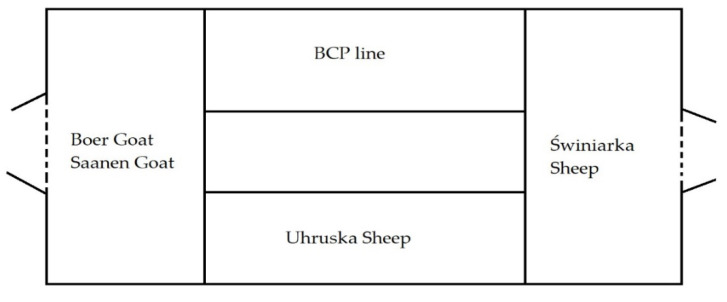
Arrangement of animals in the building (arrangement of pens in the building).

The study was conducted in the experimental station Bezek belonging to the University of Life Sciences in Lublin, located in the south-eastern part of Poland. The animals were kept in one building under uniform environmental conditions (with communicate airway) (Figure 1). Both sheep and goats are kept in the following systems: indoor (from September to mid-May), and indoor with grazing (from mid-May to early September). The animals were of the same age—4 years old, and of the same sex—females. The animals did not show any disease symptoms. All animals housed in the sheepfold were fed in the same way using the feed available on the farm. The basis of nutrition was hay, haylage and concentrated fodder (Crushed oats 0.5 kg; Rape extracted meal 0.5 kg; Hay 0.8 kg; and Green silage 2.5 kg—sheep/day).

### 2.2. Sample Collecting

The study was carried out three weeks before the animals were left on the pasture.

The samples were collected within 24 h individually from each animal in the area of the left shoulder blade, after prior careful removal of the hair. Skin swabs were collected using sterile brushes, placed in test tubes with sterile water, and then frozen at −26 °C until analysis (20 days).

### 2.3. Bacteria DNA Isolation

DNA isolation from swabs was performed using the Genomic Bacteria AX Mini kit (A&A Biotechnology, Gdansk, Poland). Then, the quality of the performed DNA isolations was checked using the Thermo Scientific NanoDrop 2000 device (Spectrophotometer, Thermo Fisher, Waltham, MA USA). The average DNA content of the samples was 30–40 μg/μL (in 50 μL). The contamination was at the level of 260/230 (contamination related to, among others, reagents used for isolation): 2.0–2.2, and 260/280 (contamination with substances such as enzymes, inhibitors): 1.8–2.0 (correct levels, according to the instruction manual of the device).

### 2.4. Real Time PCR

Real-time PCR analysis was performed with the use of a Bio-Rad CFX Connect 96 Touch apparatus with the SsoAdvanced™ Universal SYBR^®^ Green Supermix kit (Bio-Rad Laboratories, Inc., Irvine, CA, USA) at a volume of 10 μL in 3 technical repetitions (Table 1). A no template control (NTC—without DNA sample, only primers and water with PCR mix) test was additionally performed for each amplicon. The real-time PCR analysis strategy was based on the amplification of specific amplicons for the tested phyla (Firmicutes, Actinobacteria and Proteobacteria) against the reference amplicon for all bacteria (16S rDNA). The references were universal eubacterial amplicons (Table 2). In addition, the obtained results were compared to the sample constituting the calibrator with the lowest level of the studied phyla and the lowest level of the reference amplicon in order to determine the relative level of DNA in terms of the tested amplicons—Table 2.

A standard curve was performed for the genes tested to determine the efficiency of each gene. A sample dilution of 10^−3^ from the 10^−2^ to 10^−7^ series of dilutions was selected for analysis. The analysis was performed according to a protocol of 40 cycles: polymerase activation and DNA denaturation 95 °C (3 min), denaturation 95 °C (15 s), annealing 60.5 °C (15 s), extension and plate reading at 72 °C (40 s). The analysis of the melting curves for the samples was performed at temperatures ranging from 65 °C (5 s) to 95 °C (0.5 °C increments in 2 s). The data were then compiled using the CFX Maestro software (Bio-Rad Laboratories, Inc., Irvine, CA, USA). The efficiency of individual amplicons was correct (according to the standards established by BIO-RAD) and amounted to 89.4% for Firmicutes, 99.9% for Bacteroidetes, 91.6% for Actinobacteria, 94.2% for Proteobacteria and 98.4% for Universal primer.

The data were then processed using the CFX Maestro software (Bio-Rad Laboratories, Inc., Irvine, CA, USA), where the sample with a DNA quantity of 30 μg/μL and impurity levels compliant with the above-mentioned standards was an arbitrary calibrator. Using the CFX Maestro program, the levels of the tested bacteria were calculated in relation to the amount of the reference amplicon template and the differences at the level of the studied amplicons of phyla—DNA level (ΔΔCq), taking into account the amplification efficiency of individual amplicons.

### 2.5. Statistical Analysis of the Results

The obtained results were analyzed using the Statistica ver. 13.1 (StatSoft Inc., Tulsa, OK, USA). Data distribution was checked with the Shapiro–Wilk test. It was shown that the data had a normal distribution for Firmicutes; therefore, a one-way ANOVA test was performed, while the remaining data (no normal distribution: Bacteroidetes, Actinobacteria and Proteobacteria) were tested using the Kruskal–Wallis test (*p* ≤ 0.05). The differences for ANOVA were determined using Tuckey’s test (*p* ≤ 0.05). Correlations (Spearman) were also estimated in order to determine the relationship between the tested bacterial phyla (*p* ≤ 0.05).

## 3. Results

Analyzing the obtained data from real-time PCR (Table 3), significant differences were shown between some bacterial phyla. Significant differences were found in the Firmicutes level between the study groups. The highest level was found on the surface of the Saanen goat skin, while the lowest was on the surface of the skin of the BCP line sheep (1.24 and 0.09 ΔΔCq, respectively). However, in the case of the Bacteroidetes group, no significant statistical differences were found, although the highest level of this group was found on the surface of the Saanen goat skin and the lowest on the skin of the Świniarka sheep (0.73 and 0.04 ΔΔCq, respectively).

In the case of the Actinobacteria group, there were significant differences between the BCP line and the goats. The BCP line was characterized by the lowest level of these bacteria, while the Saanen goats by the highest level, and quite similar, slightly lower, was noted in Boer goats (0.25, 0.704 and 0.703, respectively). The Proteobacteria phylum generally appeared on the skin surface at the lowest level among the bacteria tested. The lowest level was found on the skin of sheep of the BCP line, while the highest in Boer goats (0.00006 and 0.256 ΔΔCq, respectively). There were also differences in the level of some studied groups between sheep and goats in general terms—Actinobacteria (*p* ≤ 0.01) and Proteobacteria (*p* ≤ 0.05).

Subsequently, a correlation (Spearman) analysis was performed. The results obtained showed that there is a correlation between some bacterial phyla. A highly statistically significant (*p* < 0.001) and positive correlation occurred between the phyla of Actinobacteria and Proteobacteria (rs = 0.5968). A highly statistically significant (*p* < 0.01) and also a positive correlation was found between the Proteobacteria and Bacteroidetes phyla (rs = 0.5158). There was also a statistically significant (*p* < 0.05) and positive correlation between the Firmicutes and Bacteroidetes phyla; however, the lowest one compared to those mentioned earlier—rs = 0.3820.

Individual differences between the females participating in the study were also analyzed. As can be observed, animals differed in the ratio of the studied phyla within their groups. The smallest differentiation was observed in Świniarka sheep, while quite a large differentiation of the studied phyla on the skin surface was observed in goats, especially in the Boer breed (data of relative ratio in Supplementary Materials).

## 4. Discussion

The skin microbiome plays a key role in keeping an animal healthy, as does the digestive tract microbiome. Its dysbiosis negatively affects the health status of the animal and can cause the growth of pathogenic microorganisms, thus being predisposed to diseases [13,14]. According to recent studies, the skin microbiome changes and is formed with age, and adult individuals are characterized by some stability [2,15]. However, despite generally recognized stability, factors such as the environment and genetics may influence its quantitative and qualitative composition [3].

In all cases, goats were characterized by higher levels of selected phyla of bacteria. However, statistical confirmation was obtained only for the participation of Actinobacteria and Proteobacteria. On the other hand, when analyzing the obtained results at the breed level, the greatest differences were found in the Firmicutes phylum, which is also considered to be one of the most numerous phyla on the surface of the skin of mammals [1]. In the case of other phyla, they remained at a fairly similar level in sheep. However, in goats, with the exception of the Actinobacteria group, the level differed quite significantly. The obtained results may be related to the occurrence of different types of hair cover in sheep and goats. In the case of sheep, it is wool, the task of which is to maintain adequate thermal insulation of the skin; additionally, a suint is deposited on the skin, which can constitute from 3 to 30% of the weight of wool [5,16]. Due to the content of fatty acids, the suint has bacteriostatic properties [17], which could have resulted in a lower and less differentiated level of the studied groups in sheep compared with goats. However, it can be suggested that the differences in the Firmicutes group between the studied sheep breeds were related to the structure of the coat as well as breed factors. The BCP line is characterized by poor yield and staple length of wool compared with Świniarka sheep or Uhruska sheep [18,19,20,21], as well as generally the lowest level of all phyla; in addition, it was created as a breed used for meat and is a synthetic line, i.e., produced by crossing several breeds and selecting the resulting population in a desirable direction [18]. The breed of animals is related to their selection to suit their living conditions, to which their skin microbiome could also adapt [22,23,24,25].

The results obtained may also be related to the withers height of the animals, as the level of Proteobacteria phylum decreased with the increased height of the individuals. Lower animals, such as Świniarka sheep in the case of sheep and Boer in the case of goats, were characterized by a lower level of Proteobacteria within the species. The tallest sheep in this experiment was the Uhruska breed, followed by the Świniarka and the BCP line [26]. Proteobacteria belong to the group of core bacteria, mainly of the digestive system—where they are also found in the highest number [27,28,29]. Therefore, it can be suggested that, depending on the height of the sheep and goats, different levels of this phylum could occur on the skin.

The thesis that most of the bacteria from the Proteobacteria group on the animal skin may come from contaminants from the ground is confirmed by the fact that the goat skin microbiome was characterized by a significantly higher abundance. This relationship may be due to the differences in the hair cover between sheep and goats. Goat hair protects the skin less from contamination from the ground than sheep wool.

The obtained species and breed differences observed in our research may also result from the physicochemical differences of the skin surface, as they may have exacerbated the variations in humidity, temperature, composition of antimicrobial peptides (AMP) and lipid content. In addition, communication between surface microbes and deeper layers of skin cells is suspected, which is also associated with the physicochemical indicators of the skin; however, further research is required [30,31,32,33].

In addition, the large SD coefficients for each breed group indicated a large individual variability, suggesting, as in the studies on the digestive system, the influence of an individual on the microbiological composition of an organism. The microbiome has recently been considered an individual feature of a given organism. Studies on mice have shown the influence of host genetics on the development of an individual composition of intestinal microorganisms in these animals [34,35]. However, this aspect requires further research due to an insufficient amount of research carried out so far.

Correlation has also been demonstrated between individual bacterial phyla. Statistically significant and positive correlations were demonstrated between Actinobacteria and Proteobacteria, Proteobacteria and Bacteroidetes as well as Firmicutes and Bacteroidetes. The correlations obtained may indicate a kind of cooperation between the above-mentioned phyla, which confirms the interaction of the microbiome on the skin [36,37].

## 5. Conclusions

The differences observed from the RT PCR analyses in the level of selected bacterial phyla could result from the differences in the size of the animals (body weight, height and size of a given breed), as well as the influence of the individual on the microbiome composition. In research, this has also been demonstrated through correlations to better understand the functioning of the skin microbiome in the future. However, the obtained results require further research in order to determine the interaction of the skin microbiome.

## Figures and Tables

**Table 1:** Mix Ratio to PCR.

Component	Volume in 10 μL Reaction
SsoAdvanced™ Universal SYBR^®^ Green Supermix	5 μL
Primer (F + R)	1 μL (0.8 μM)
DNA	2 μL (0.04–0.015 × 10^−4^)
Sterile water	2 μL

**Table 2:** Primers used in RT PCR analysis.

Name	Forward (5′-3′)	Reverse (5′-3′)	Source
Universal Eubacterial Genes	530F (5′-GTC CCA GCM GCN GCG G)	1100R (5′-GGG TTN CGN TCG TTG)	[9]
Firmicutes	928F-Firm (5′-TGA AAC TYA AAG GAA TTG ACG)	1040FirmR (5′-ACC ATG CAC CTG TC)	[10]
Actinobacteria	Act1159R (5′-TCCGAGTTRACCCCGGC)	Eub338F ACGGGCGGTGTGTACA	[11]
Proteobacteria	27F (5′ GAGTTTGATCMTGGCTCAG-3′)	1529R (5′ CAKAAAGGAGGTGATCC-3′)	[12]

**Table 3:** DNA level (ΔΔCq) of selected bacterial phyla on the skin of animals (a,b,c,d—*p* ≤ 0.05; A,B—*p* ≤ 0.01—breed/species differences; ** *p* ≤ 0.01, * *p* ≤ 0.05—species differences).

Breed/Species		Świniarka Sheep	Uhruska Sheep	BCP Line	Saanen Goat	Boer Goat	Sheep	Goat
Firmicutes	Average	0.998 ^A,a^	0.7874 ^d^	0.0916 ^B,c^	1.2415 ^d^	0.3307 ^b^	0.625423	0.786096
	SD	0.514	0.1998	0.0114	0.8913	0.1317	0.515724	0.817944
Bacteroidetes	Average	0.0354	0.47258	0.112967	0.72712	0.33385	0.206970	0.530481
	SD	0.0789	0.5379	0.0968	0.7954	0,2354	0.382145	0.646131
Actinobacteria	Average	0.1827	0.4447	0.0253 ^a^	0.7044 ^b^	0.7034 ^b^	0.217563 **	0.703883 **
	SD	0.2471	0.4096	0.0259	0.5684	0.3361	0.335674	0.487704
Proteobacteria	Average	0.0002 ^a^	0.00017 ^a^	0.00006 ^a^	0.0320 ^a^	0.2558 ^b^	0.000153 *	0.143916 *
	SD	0.0003	8.1854 × 10^5^	7.1181 × 10^5^	0.0382	0.2845	0.000255	0.242140

SD: standard deviation.

## Data Availability

The data presented in this study are available upon request from the corresponding author. The data are not publicly available due to privacy.

## Supplementary Materials

The following are available online at https://www.mdpi.com/article/10.3390/ani11092734/s1, Figures S1–S5—Relative Individuals Differences.
