# Supplementary material for: The Level of Selected Bacterial Phyla on the Skin Surface of Small Ruminants According to the Breed and Species
Source: Animals (Basel). 2021 Sep 18;11(9):2734. doi: 10.3390/ani11092734 (PMC8472796; doi:10.3390/ani11092734)
Supplement: Supplementary file 1 [file animals-11-02734-s001.zip › animals-1323957-supplementary.pdf]

Data of relative ratio (%) of selected bacteria phyla:

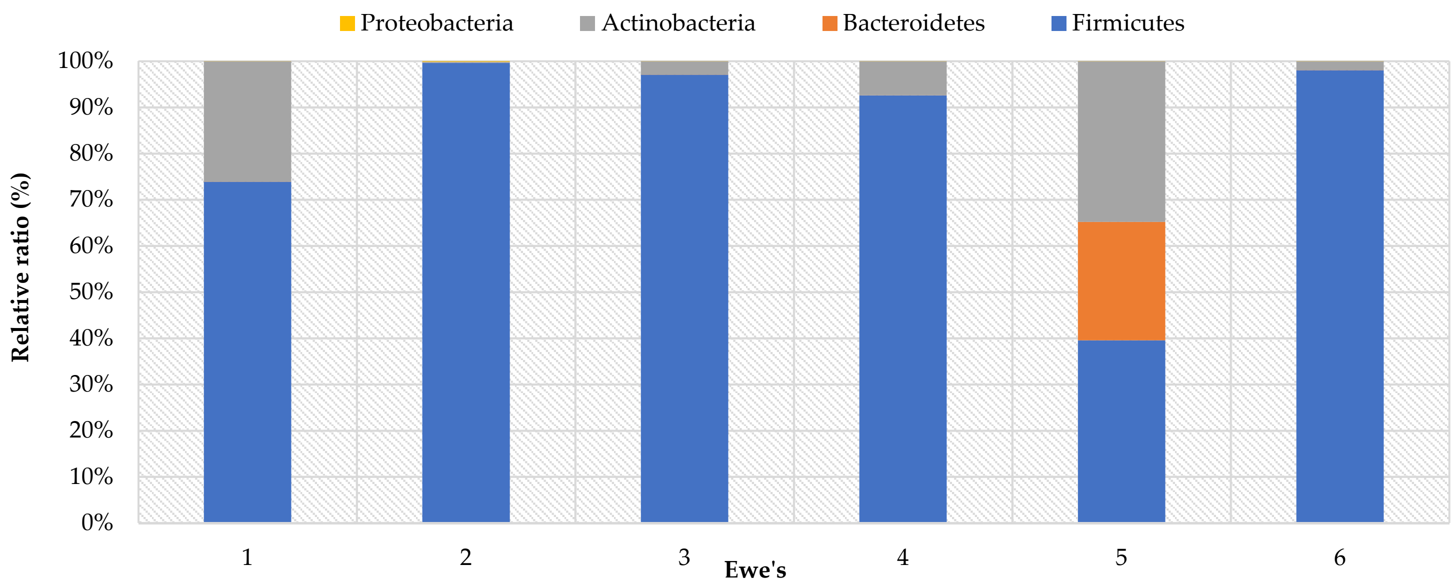

Figure S1. Relative ratio (%) of selected phyla on Świniarka sheep skin surface.

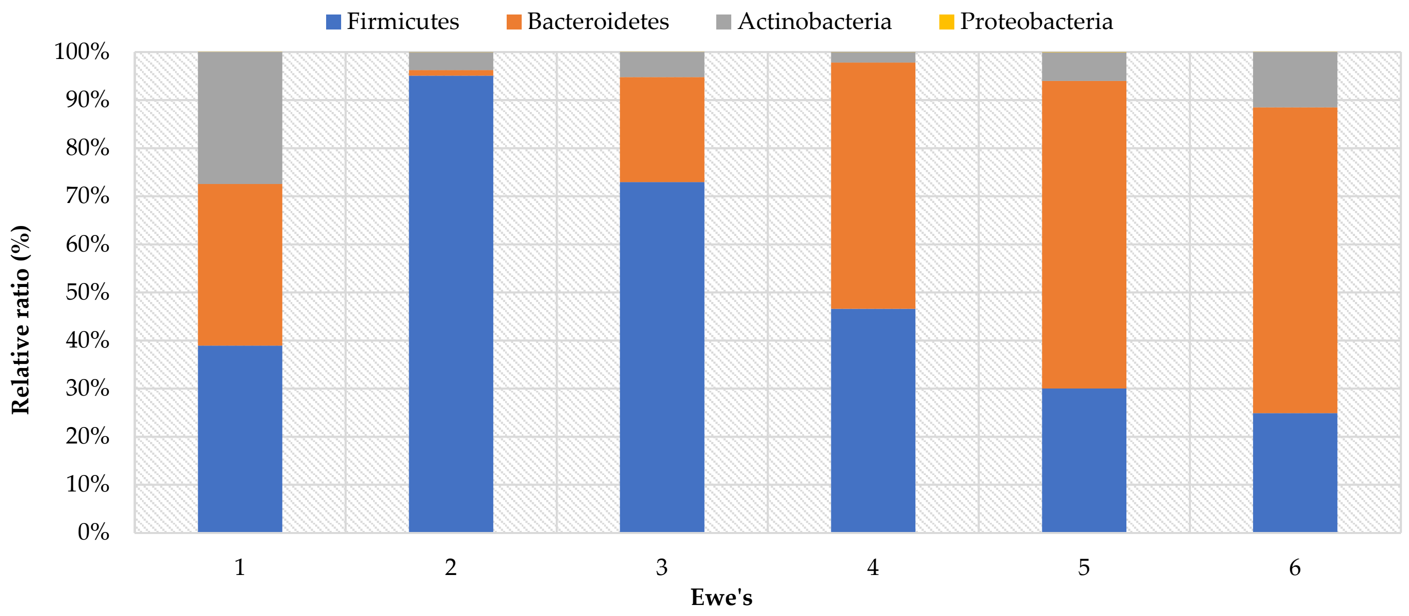

Figure S2. Relative ratio (%) of selected phyla on BCP sheep skin surface.

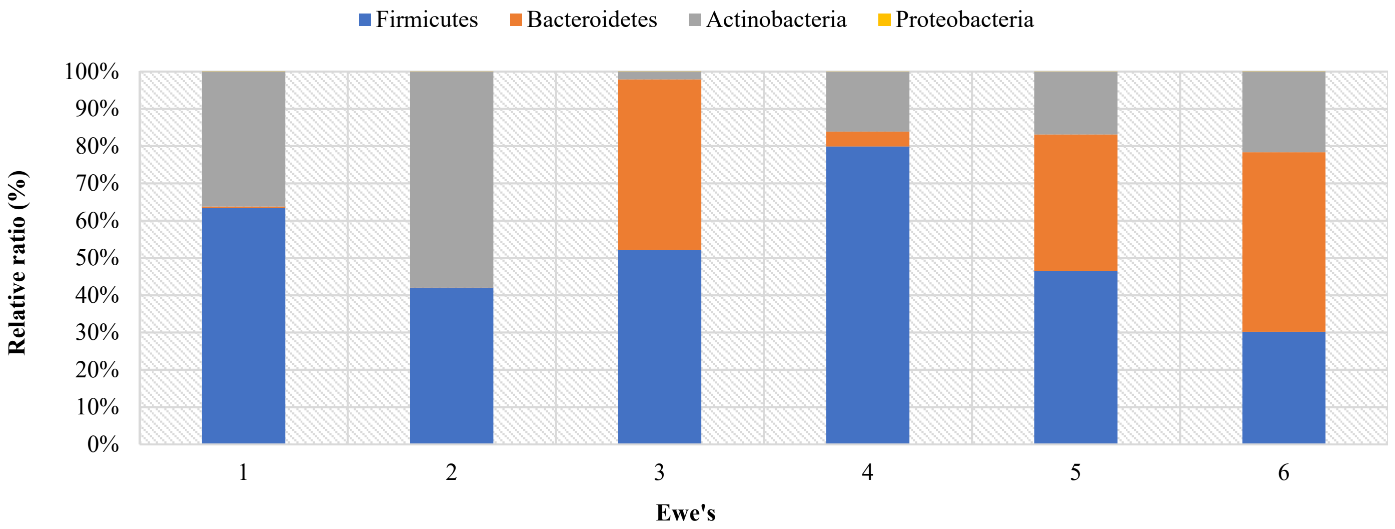

Figure S3. Relative ratio (%) of selected phyla on Urhuska sheep skin surface.

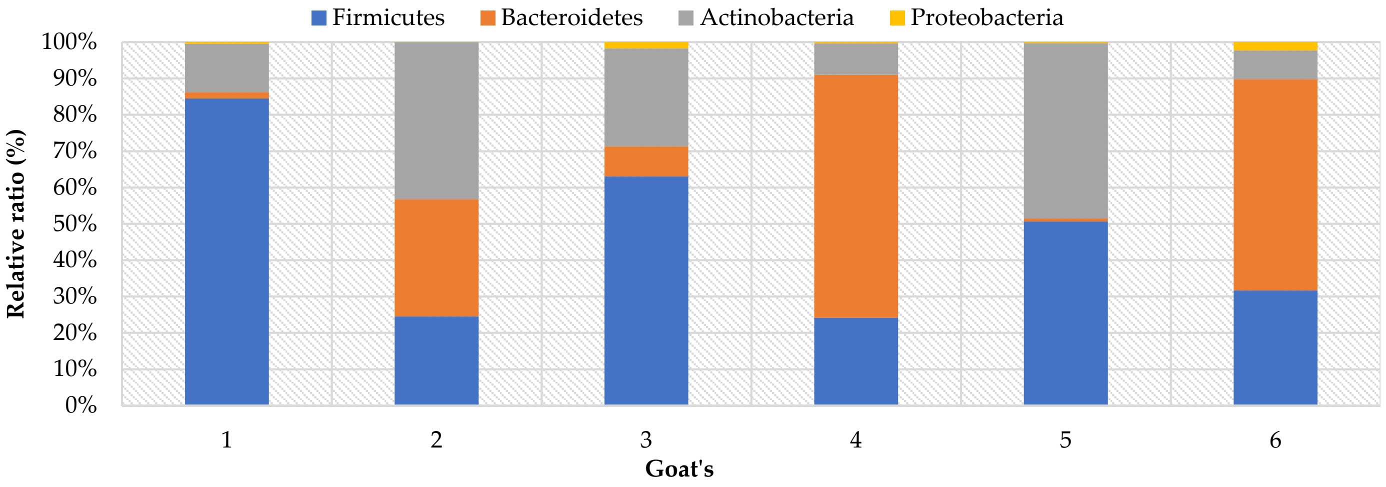

Figure S4. Relative ratio (%) of selected phyla on Saanian Goat skin surface.

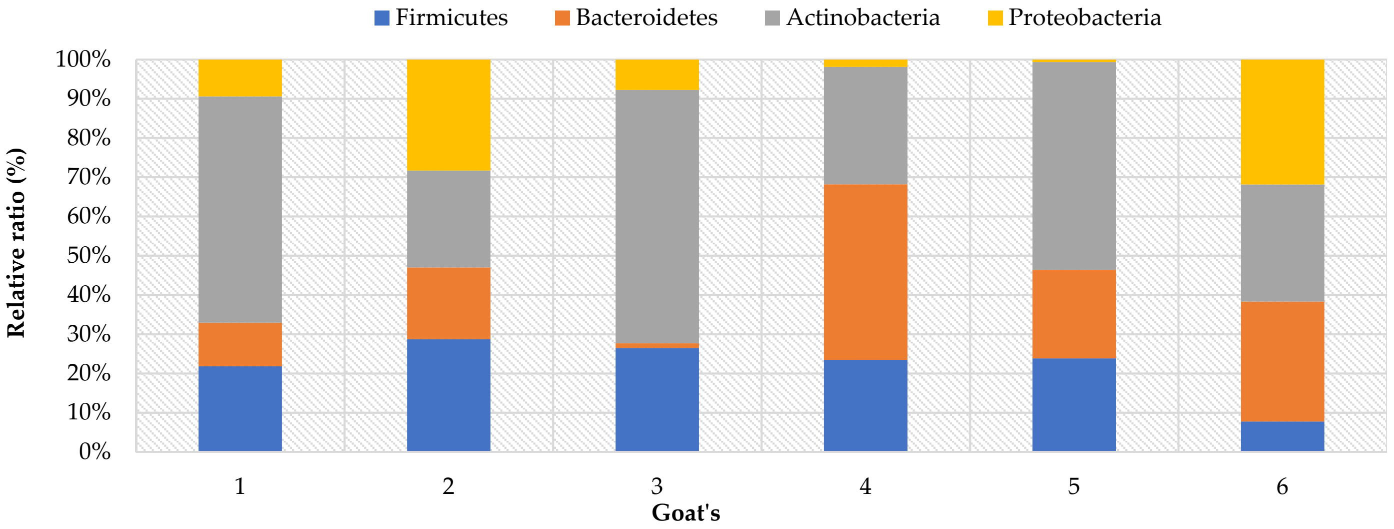

Figure S5. Relative ratio (%) of selected phyla on Boer Goat skin surface.
